# An EEG-Based Edge-AI Framework for Alzheimer’s and Creutzfeldt–Jakob Disease Classification

**DOI:** 10.3390/s26103274

**Published:** 2026-05-21

**Authors:** Muhammad Suffian, Cosimo Ieracitano, Nadia Mammone, Angelo Pascarella, Edoardo Ferlazzo, Francesco Carlo Morabito

**Affiliations:** 1 DICEAM, Mediterranea University of Reggio Calabria, 89125 Reggio Calabria, Italy; nadia.mammone@unirc.it (N.M.); morabito@unirc.it (F.C.M.); 2 DICMaPI, University of Naples “Federico II”, 80125 Naples, Italy; cosimo.ieracitano@unina.it; 3 Department of Medical and Surgical Sciences, Magna Græcia University of Catanzaro, 88100 Catanazaro, Italy; a.pascarella@unicz.it (A.P.); ferlazzo@unicz.it (E.F.); 4 Regional Epilepsy Centre, Great Metropolitan “Bianchi-Melacrino-Morelli” Hospital, 89124 Reggio Calabria, Italy

**Keywords:** Alzheimer’s disease (AD), Creutzfeldt–Jakob disease (CJD), electroencephalography (EEG), brain-computer interface, convolutional neural networks, transformers, edge-AI, green AI

## Abstract

Electroencephalography (EEG) has emerged as a promising non-invasive tool for the diagnosis of neurodegenerative disorders, and artificial intelligence (AI) has shown significant potential in this domain, as demonstrated by recent studies. However, strong inter-subject variability remains a major challenge, limiting the ability of AI-based models to learn disease-specific features that generalize across individuals, thereby hindering the development of clinically deployable subject-independent systems. In this work, we propose a cross-subject, AI-based EEG classification framework to distinguish between Alzheimer’s disease (AD), Creutzfeldt–Jakob disease (CJD), and healthy control subjects using clinical EEG data collected from a local hospital. A lightweight hybrid deep learning model is developed, combining a two-layer one-dimensional convolutional neural network with a two-layer Transformer encoder to capture both local temporal patterns and long-range dependencies in EEG signals. The proposed model achieves an average classification accuracy of 97%, representing a 3% improvement over a baseline model evaluated on a cohort of 36 subjects. To assess deployment feasibility in real-time clinical settings, the trained model is implemented and evaluated on an edge-AI platform (NVIDIA Jetson AGX Orin), demonstrating energy efficiency for the inference with a compact model footprint. These results indicate that the proposed approach provides an accurate, efficient, and practically deployable solution for subject-independent EEG-based classification of neurological disorders.

## 1. Introduction

Neurodegenerative disorders present an increasing burden on healthcare systems worldwide, owing to their progressive course and the limited availability of accessible diagnostic tools suitable for large-scale screening and early differential diagnosis. Alzheimer’s disease (AD) typically follows a slow progression as the leading cause of dementia, whereas Creutzfeldt–Jakob disease (CJD) presents as a rare, rapidly progressive dementia with clinical symptoms that often overlap with AD [1,2]. Discriminating between AD and CJD is critical in the context of large-scale screening, as the two conditions differ markedly in prognosis, progression rate, and clinical management despite overlapping early cognitive symptoms. Current diagnostic procedures often rely on costly neuroimaging techniques, which limit their ability in routine clinical practice and are not suitable for large-scale screening. In this context, electroencephalography (EEG) has gained increasing attention as a relatively low-cost, non-invasive, and widely deployable modality for supporting neurological disorder diagnosis [3,4,5].

EEG-based analysis has demonstrated potential in capturing disease-related alterations in brain dynamics associated with neurodegeneration. Early studies primarily relied on handcrafted spectral and connectivity features combined with conventional machine learning classifiers [6]. Mammone et al. [7] introduced a permutation disalignment index (PDI) for differential diagnosis of mild cognitive impairment (MCI) and AD patients using EEG data. The authors reported an increase in the PDI value in the delta and the theta bands of patients with MCI, converted subsequently to AD. Their finding shows that changes in EEGs can be observed before the onset of clinical symptoms. Subsequently, Mammone et al. [8] investigated the permutation Jaccard distance (PJD) to quantify the brain electrical connectivity changes over a longitudinal evaluation of MCI patients. They reported that four patients with MCI converted to AD presented an increase in the PJD value in the delta and the theta bands, while the other patients did not exhibit such an increase. Amezquita-Sanchez et al. [9] presented an automated methodology for MCI and AD diagnosis using advanced signal processing (MUSIC-EWT) to extract features from brainwaves and achieved an accuracy around 90%. More recently, deep learning approaches, particularly convolutional neural networks (CNNs), have been introduced to automatically learn discriminative representations from EEG signals [10,11,12]. An auto-encoder-based deep learning methodology [13] was proposed to differentiate early-stage CJD and other forms of rapidly progressive dementia (RPD); with fine-tuning the parameters of the globally trained model, it was able to achieve an average accuracy of 89% to differentiate CJD and RPD, and similar results were achieved for CJD versus AD and CJD versus healthy controls (CNTRL). Transformer-based architectures have further shown promise in modeling long-range temporal dependencies in time-series data [14]. ADformer, an end-to-end representation learning model, was proposed with spatial–temporal transformer to learn the multi-granularity of spatial and temporal features from raw EEG signals [15]. ADformer was tested on four datasets and it achieved a maximum F1-score of 92.89%. Despite these advances, many reported results rely on experimental protocols that do not reflect realistic clinical deployment scenarios.

A practical challenge that is often overlooked in EEG studies is the presence of artifactual and corrupted segments in real clinical recordings. EEG data acquired in hospital environments are frequently affected by electrode detachment, patient movement, or acquisition interruptions, resulting in non-stationary artifacts and abrupt signal cuts [16]. Such issues are rarely present in public benchmark datasets but are unavoidable in real-world clinical practice. Failure to explicitly handle these discontinuities can negatively impact downstream learning and model reliability. A major limitation in existing EEG-based diagnostic studies is the widespread use of cross-validation strategies at trial-level rather than subject-level, causing train–test contamination as trials from the same subject may appear in both training and testing sets. Such evaluation protocols can lead to subject leakage and overly optimistic performance estimates, thereby limiting generalization to unseen subjects [17]. Subject-independent validation strategies, such as leave-one-subject-out (LOSO) cross-validation, are therefore increasingly recognized as essential for reliable assessment of EEG-based classification systems [11]. When the LOSO approach is adopted, the model is trained using data from N-1 subjects and validated using data from the remaining subject. However, achieving robust performance under LOSO constraints remains challenging due to strong inter-subject variability, particularly for small and heterogeneous clinical datasets. In parallel, there is growing interest in deploying deep learning models directly at the point of care using edge-AI platforms. Edge-based inference enables low-latency, privacy-preserving, portability and reliable operation without continuous reliance on cloud infrastructure, which is particularly desirable in clinical environments [18]. However, many state-of-the-art EEG deep learning models are computationally intensive and unsuitable for embedded devices. This has motivated increasing attention toward compact architectures and green-AI principles that emphasize energy efficiency and reduced computational complexity while maintaining strong predictive performance [19].

In this work, a cross-subject EEG classification framework is proposed for distinguishing AD, CJD, and haelthy control subjects using real clinical EEG data collected from a local hospital. The dataset comprises 36 subjects (12 per class) and includes recordings with known signal artifacts (labelled by expert clinicians), which are explicitly removed using provided temporal annotations prior to analysis. Clean EEG signals are segmented into 5-second trials and evaluated using a LOSO cross-validation strategy to prevent subject leakage. A lightweight hybrid deep learning model is developed, which integrates a two-layer one-dimensional CNN with a two-layer Transformer encoder to capture both local temporal patterns and long-range dependencies in EEG signals. Furthermore, to assess edge feasibility, the trained model is implemented and evaluated on an NVIDIA Jetson AGX Orin edge-AI platform [20], demonstrating efficient inference with a compact model footprint. The model is intentionally designed with green-AI considerations, aiming to balance classification performance with computational efficiency.

The main contributions of this work are:

1. The development of a compact CNN–Transformer architecture which achieves high classification accuracy;
2. The adoption of a rigorous LOSO evaluation protocol to ensure subject-independent validation and reliable performance assessment;
3. The deployment of the developed framework on an edge-AI device towards an EEG-based diagnostic tool suitable for real clinical settings;
4. To support Open Science, the source codes are available on a dedicated GitHub repository (https://github.com/AI-Lab-UniRC/FAIR-NAEL-Database/tree/main/EEG (accessed on 7 April 2026)).

This paper is organized as follows: Section 2 describes the materials and methods, including the EEG signal processing, the proposed EEGDecoder framework, and the experimental setup; Section 3, Results, presents the experimental results, highlighting the performance of EEGDecoder and the potential of the proposed framework and its deployability on edge-AI; and Section 4 concludes this paper with future work considerations.

## 2. Materials and Methods

**Figure 1:**
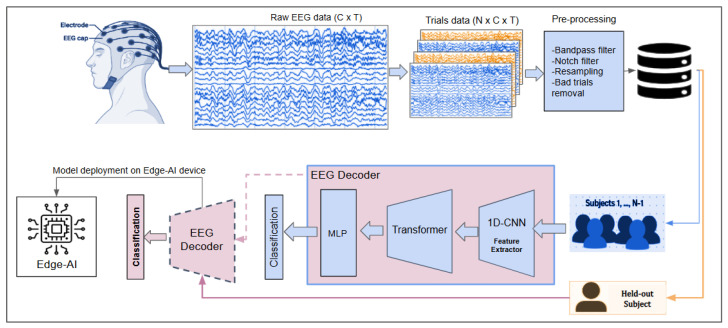
Overview of the proposed hybrid EEG decoding and classification framework. The upper part illustrates the EEG data preprocessing and dataset construction pipeline. Raw multichannel EEG recordings from a single subject are shown as channel-by-time signals (C×T). EEG recordings are then segmented into non-overlapping 5-second trials, forming number of trials × channels × time (N×C×T), which are stored as the subject-wise dataset. The lower part depicts the cross-subject classification framework using a leave-one-subject-out (LOSO) strategy, where the model is trained on data from N−1 subjects and evaluated on the held-out subject. The proposed hybrid model consists of a one-dimensional convolutional neural network (1D-CNN) for feature extraction, followed by a Transformer encoder and a multilayer perceptron (MLP) for classification. Finally, the trained EEG decoder is deployed on an edge-AI device to evaluate inference performance and deployment feasibility.

The proposed methodology is illustrated in Figure 1, in which the top panel shows the EEG data acquisition and preprocessing, and the bottom panel shows the hybrid model and its training and deployment on edge-AI. The EEG data consist of multichannel clinical recordings from subjects diagnosed with Alzheimer’s disease (AD), Creutzfeldt–Jakob disease (CJD), and healthy controls. The EEG recordings of size C×T were obtained. These signals were then segmented into non-overlapping 5-second trials, resulting in trials of size N×C×T, which were preprocessed and organized on a subject-wise basis to construct the final dataset.

The extracted EEG trials were processed using a hybrid deep-learning-based classification model, referred to as EEG Decoder. The proposed decoder integrates a lightweight one-dimensional convolutional neural network (1D-CNN) for local temporal feature extraction, followed by a Transformer encoder to model long-range temporal dependencies in the EEG signals. The final classification stage is performed using a fully connected layer to distinguish between AD, CJD, and control subjects. To ensure a realistic and cross-subject evaluation, a LOSO cross-validation strategy was employed. Finally, to assess deployment feasibility in real-world clinical settings, the trained EEG decoding model was deployed on an edge-AI platform, where inference performance and computational efficiency were evaluated. This design choice supports the development of compact and energy-efficient EEG-based diagnostic systems suitable for real-time and resource-constrained clinical environments.

The proposed methodology is described in the following subsections: (Section 2.1) EEG signal preprocessing and dataset construction; (Section 2.2) the proposed hybrid CNN-Transformer strategy; and (Section 2.3) experimental setup, which includes cross-subject EEG decoding and performance evaluation hyperparameters.

### 2.1. EEG Signal Preprocessing and Dataset Construction

In this study, a private clinical EEG dataset consisting of recordings from 36 subjects was collected at the Unit of Neurology of the Great Metropolitan ‘Bianchi-Melacrino-Morelli’ Hospital in Reggio Calabria. The dataset comprises three groups: Alzheimer’s disease (AD), Creutzfeldt–Jakob disease (CJD), and healthy controls (CNTRL), with 12 subjects per group. The clinical diagnosis of participants was performed by specialized neurologists. AD patients were assessed following current consensus criteria [21,22]. Diagnosis of sporadic CJD was confirmed based on updated diagnostic criteria, utilizing a combination of clinical symptoms, EEG patterns, and specialized biomarker evidence [23]. EEG signals were recorded using 19 electrodes positioned according to the 10–20 international system, with Cz serving as the ground electrode (reference electrode). The recordings were originally acquired under clinical conditions, resulting in variations in recording duration across subjects depending on diagnosis and clinical protocol. All recordings were performed under controlled, eyes-closed and awake status to minimize artifacts. The EEG signals were sampled at a rate of 256 Hz (resampled for one subject from 512 Hz to 256 Hz); the signals were band-pass filtered between 1.6 Hz and 40 Hz, and notch-filtered at 50 Hz to suppress power-line interference.

The control cohort subjects had an unremarkable clinical history and normal findings on instrumental examinations. The study cohort (N=36) exhibited a mean age of 68.24 years (SD=9.27), with ages ranging from 55.66 to 83.59 years. A Shapiro–Wilk test for normality confirmed that the age distribution did not significantly deviate from a normal distribution (W=0.918,p=0.209). The cohort primarily represents a late-middle-aged-to-elderly demographic, consistent with the typical onset window for neurodegenerative pathologies such as AD and CJD. The raw EEG recordings contained signal discontinuities and corrupted segments resulting from clinical acquisition artifacts, such as temporary electrode detachment or recording interruptions. The start and end times of these discontinuities were identified by expert EEG operators using the EEG recording machine; this information was provided for each subject along with the EEG data and we used this information to remove the affected signal segments by writing a Python v.3.12 script to automate the removal of affected segments. While the raw recording lengths varied across the longitudinal window due to clinical constraints, standardization was achieved through rigorous artifact rejection and epoch selection. Following the removal of ocular and myogenic artifacts via Independent Component Analysis (ICA), we extracted 40 non-overlapping trials of 5 s each, and an equal number of trials was extracted for each subject to ensure class balance across AD, CJD, and CNTRL groups. This resulted in a standardized dataset of 3.33 min of ‘clean’ signal per participant. ICA was applied using the Python MNE framework (https://mne.tools/stable/index.html (accessed on 7 April 2026)) with the number of components selected to preserve 95% of the signal variance (n_components = 0.95) and a fixed random seed (as set for the whole experiment, e.g., 2025) to ensure reproducibility. Artifact-related ICA components were identified automatically using correlation-based EOG detection provided by the MNE toolbox. The detected ocular-related components were excluded before reconstructing the cleaned EEG signals. However, due to artifact rejection during EEG preprocessing, one subject in the CJD class had only 17 usable trials. To preserve data integrity, we chose not to perform data augmentation or artificially inflate the number of trials for this subject. The reduced number of trials was intentionally retained in its original form, as under the LOSO-CV evaluation framework it introduces only a negligible impact on the training stage. The remaining 35 subjects each contribute 40 trials, providing sufficient data for robust model learning. Furthermore, evaluating the held-out subject using its available clean trials does not violate the subject-independent evaluation protocol. The resulting trials were organized on a subject-wise basis and used for subsequent model training and evaluation. It is to be noted that all data were anonymized prior to analysis in accordance with data protection requirements. The dataset is available at GitHub (https://github.com/AI-Lab-UniRC/FAIR-NAEL-Database/releases/tag/EEG_AD_NAEL (accessed on 7 April 2026)).

### 2.2. Proposed Hybrid EEG Decoder System

**Figure 2:**
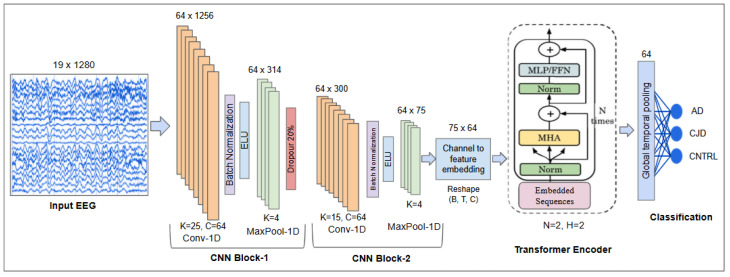
Architecture of the proposed hybrid model. Raw EEG trials are processed by a 1D-CNN for local temporal feature extraction, followed by a Transformer encoder to model long-range dependencies. The output is aggregated via temporal pooling and classified using a fully connected layer.

The proposed EEG-based hybrid CNN-Transformer decoding model, EEG Decoder, is illustrated in Figure 2. The main components of decoding model are summarized as follows:

1. 1D CNN as a feature extractor: input EEG trials of size C×T are fed into the custom 1D-CNN feature extractor.
2. Sequence creation: CNN feature maps are permuted and reshaped to form sequences of embeddings compatible with the Transformer encoder input.
3. Transformer encoder learning: the sequences are processed by a 2-layer Transformer encoder with 2 attention heads.
4. Temporal pooling and classification: The Transformer output is aggregated along the temporal dimension using mean pooling, producing a fixed-size embedding vector. This vector is passed through a fully connected (MLP) layer to classify each EEG trial into one of the three classes: AD, CJD, or CNTRL.

The components of EEGDecoder are described in detail in the following subsections.

#### 2.2.1. Custom 1D-CNN Feature Extractor

The CNN feature extractor is designed to capture local temporal patterns from multichannel EEG signals. The CNN consists of two 1D convolutional layers with Exponential Linear Unit (ELU) activations, batch normalization, and max-pooling, producing high-level temporal feature maps. The first convolutional layer uses $n_{\mathrm{in}}=19$ channels, and $n_{\mathrm{out}}=64$ channels with kernel size K=25, followed by batch normalization, ELU activation, and max-pooling (K=4). The second convolutional layer has 64 channels with kernel size K=15, batch normalization, ELU, and max-pooling (K=4). After the second pooling layer, the feature maps have size 64×75 (channels × temporal steps) and are permuted to sequences of shape 75×64 for the Transformer encoder.

#### 2.2.2. Transformer Encoder

The Transformer encoder is employed to capture long-range temporal dependencies in EEG signals, which are often difficult to model using convolutional operations alone. By leveraging self-attention, the encoder can dynamically assign importance to different temporal segments, enabling a global understanding of the signal. The encoder consists of two identical layers, each comprising a multi-head self-attention (MHA) mechanism followed by a position-wise feedforward network (FFN). Each sub-layer is combined with residual connections and layer normalization to improve training stability and convergence.

Given an input embedding sequence Z, three projections are computed to obtain the queries (*Q*), keys (*K*), and values (*V*). The attention mechanism evaluates the relevance between all pairs of time steps using scaled dot-product attention:

(1) $$\mathrm{Attention}\left(Q,K,V\right)=\mathrm{softmax}\left(\frac{QK^{⊤}}{\sqrt{d_{k}}}\right)V$$

where $d_{k}$ denotes the dimensionality of the key vectors. The scaling factor ensures numerical stability by preventing excessively large dot-product values. To enhance representational capacity, multiple attention heads are used in parallel. Each head captures complementary temporal relationships within the EEG sequence. The outputs of all heads are concatenated and projected back to the embedding dimension:

(2) $$\mathrm{MHA}\left(Z\right)=\mathrm{Concat}\left({\mathrm{head}}_{1},\ldots ,{\mathrm{head}}_{h}\right)W^{O}.$$

Following the attention block, each embedding is processed independently by a feedforward network applied at each time step:

(3) $$\mathrm{FFN}\left(z\right)=\mathrm{ReLU}\left(zW_{1}+b_{1}\right)W_{2}+b_{2}$$

This component introduces non-linearity and refines the learned representations by projecting them into a higher-dimensional space and back. Each sub-layer is followed by a residual connection and layer normalization, defined as follows:

y=LayerNorm(x+F(x))

where F(·) denotes either the MHA or FFN operation. This structure facilitates gradient propagation and stabilizes the training process.

#### 2.2.3. Temporal Pooling and Classification

The Transformer outputs are aggregated along the temporal dimension using mean pooling:

(4) $$p=\frac{1}{T^{\prime }}\sum_{t=1}^{T^{\prime }}z_{t}$$

which produces a fixed-size embedding vector $p\in R^{64}$. This embedding is fed into a fully connected layer for three-class classification (AD, CJD, and CNTRL). The compact size of the proposed model ensures efficiency for edge deployment while maintaining high accuracy.

### 2.3. Experimental Setup

Table 1 summarizes the training and architectural hyperparameters used for the proposed hybrid CNN–Transformer (EEG Decoder) model.

A trial-and-error strategy was carried out for finding the best performance parameters and it was observed by looking at the classification performance and computational efficiency considerations. The different model configurations were evaluated by varying key parameters such as kernel size, Transformer embedding dimension (d_model), learning rate, weight decay, and dropout rate. Particular emphasis was placed on maintaining a compact model size to support deployment on edge-AI devices and align with green AI principles. While performing trial-and-error, for the CNN feature extractor, the kernel sizes and pooling configurations were varied to balance temporal resolution and model complexity. The final CNN architecture consists of two 1D convolutional layers with kernel sizes of 25 and 15, each followed by batch normalization, ELU activation, and max-pooling with a pooling factor of 4. This configuration achieved the best trade-off between feature extraction and computational cost. The hybrid model was trained using the Adaptive Moment Estimation (Adam) optimizer [24] with a learning rate of $1\times 10^{-3}$ and a weight decay of $1\times 10^{-4}$. A batch size of 64 was used across all experiments, and training was performed for a maximum of 30 epochs. During training, early stopping was employed to save computational resources and avoid overfitting the model. Validation accuracy was used as a monitored metric for early stopping with patience = 10. The Transformer encoder was configured with 2 encoder layers and 2 attention heads to model long-range temporal dependencies while keeping the architecture lightweight. It is worth mentioning that increasing the number of layers or attention heads resulted in marginal performance gains at the expense of increased computational cost and energy consumption. Therefore, the selected configuration reflects a conscious trade-off between accuracy and efficiency. The embedding dimension of the Transformer encoder was set to 64, matching the number of output channels of the CNN feature extractor. The remaining hyperparameters of the Transformer encoder were kept at their default values.

For the comparison, we used EEGNet [10], an established baseline model for EEG decoding. It was configured using the same input time window and number of EEG channels as the proposed model. To ensure a fair comparison, EEGNet was evaluated under the exact same experimental protocol as the proposed model. Specifically, both models were trained and tested using the same LOSO cross-validation mechanism, identical data splits, and the same preprocessing pipeline. Specifically, an EEGNet-8,2 architecture was employed with $F_{1}=8$ temporal convolutional filters in the first layer to capture frequency-specific patterns, followed by a depthwise convolution with depth multiplier D=2 to learn spatial filters across EEG channels. This resulted in $F_{2}=F_{1}\times D=16$ feature maps in the separable convolution block. A kernel length of 64 samples was used for temporal filtering, and a dropout rate of 0.25 was applied to reduce overfitting. This configuration follows standard EEGNet design principles while ensuring a lightweight and computationally efficient baseline.

In LOSO cross-validation evaluation, data from N−1 subjects are used for training (global training of the model), while the left-out subject’s data are used for testing (local testing). This process is repeated *N* times (where *N* denotes the total number of subjects), ensuring that each subject serves as the test set once. Final cross-validation performance was computed as the average across all test folds. The LOSO protocol was following:

- Outer loop: LOSO splits for cross-subject evaluation:
  – In each iteration, one subject is held out for testing, and the remaining N−1 subjects’ data are used for model training and validation.
- Inner loop (model training and selection on N−1 subjects):
  – Data from the N−1 subjects are split into 5 folds, 4 folds for training and a held-out fold for cross-validation, ensuring that all trials from a given subject were contained within a single fold, thereby preventing data leakage.
  – Early stopping is applied based on validation performance.
  – The model with the best validation performance across folds is selected.
- Held-out subject testing:
  – The best saved model was loaded and held-out subjects data were used to test it.

Data normalization was performed after making the LOSO splits, and only the training part of the data was normalized and then, during testing, the same normalization parameters were used to transform the test set to avoid the data leakage. This ensures an unbiased assessment of generalization across unseen subjects.

All experiments were implemented using the PyTorch v.2.5 deep learning framework [25]. Model training was conducted on a workstation running Ubuntu, equipped with an NVIDIA RTX 4000 Ada Generation GPU, an Intel Xeon(R) CPU @ 2.30 GHz, and 125 GB of RAM.

## 3. Results

The performance of the proposed and baseline model was evaluated using standard metrics, namely accuracy, precision, recall, F1-score, and Cohen’s Kappa. Accuracy $=\frac{TP\;+\;TN}{TP\;+\;TN\;+\;FP\;+\;FN}$ measures the proportion of correctly classified samples. Precision $=\frac{TP}{TP\;+\;FP}$ reflects the proportion of true positives among predicted positives. Recall $=\frac{TP}{TP\;+\;FN}$ measures the proportion of true positives among actual positives. F1-score $=\frac{2\cdot \mathrm{Precision}\cdot \mathrm{Recall}}{\mathrm{Precision}\;+\;\mathrm{Recall}}$ provides the harmonic mean of precision and recall. Cohen’s Kappa quantifies the agreement between predicted and true labels while accounting for agreement occurring by chance [26].

For this three-class classification problem (AD vs. CJD vs. CNTRL), standard binary metrics (accuracy, precision, recall, and F1-score) were extended using a one vs rest (OvR) strategy. For each class (i), true positives $\left(TP_{i}\right)$, false positives $\left(FP_{i}\right)$, false negatives $\left(FN_{i}\right)$, and true negatives $\left(TN_{i}\right)$ were computed. Performance evaluation was conducted at both the trial level and the subject level, with primary emphasis on subject-level results due to their clinical relevance. At the trial level, accuracy was computed as the global proportion of correctly classified trials, while precision, recall, and F1-score were computed using weighted averaging, where each class is weighted by its number of true instances (support). For subject-level evaluation, trial-level predictions corresponding to each subject were aggregated using a majority voting scheme, whereby the subject was assigned the class receiving the highest number of predicted trial labels. A subject was considered correctly classified if the aggregated label matched the ground truth. The results reported in Table 2 correspond to subject-level performance, where accuracy, precision, recall, and F1-score were computed across all subjects based on these aggregated predictions. Cohen’s Kappa (κ) was also computed at the subject level to measure agreement between predicted and true labels beyond chance. This evaluation strategy ensures that each subject contributes equally to the final performance metrics, thereby avoiding bias due to unequal numbers of trials per subject. Additionally, metrics computed from the aggregated subject-level confusion matrix are consistent with those obtained using standard evaluation functions.

**Figure 3:**
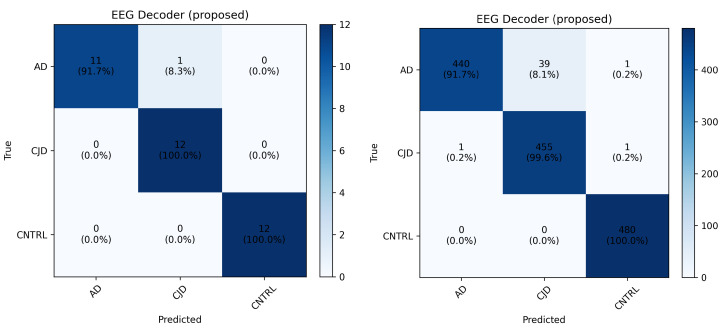
Confusion matrices produced by EEGDecoder in the AD vs. CJD vs. CNTRL classification using the LOSO approach. (**Left**) The matrix reports performance at the subject level (percentage of subjects correctly classified); and (**right**) the matrix reports performance on the trial level (percentage of trials correctly classified).

As reported in Table 2, the experimental results show that proposed EEGDecoder outperformed EEGNet across all metrics, confirming strong overall classification capability. In particular, EEGDecoder achieved an average accuracy, precision, recall, F1-score and Cohen’s Kappa of 97.08%, 94.22%, 93.45%, 93.83%, and 0.95, respectively. In contrast, EEGNet achieved lower performance across the same metrics, i.e., 93.90%, 88.72%, 85.45%, 86.70%, and 0.87, respectively. To further evaluate robustness, the experiment was repeated for the complete LOSO-CV using five different random seeds (42, 99, 123, 2025, 2026) affecting model initialization, data shuffling, and optimization (including Pytorch and Cuda initialization). Across all runs, the subject-level predictions remained consistent, resulting in similar classification outcomes for all 36 subjects; for the proposed model, the mean test accuracy and standard deviations were 97.05±0.21, and for EEGNet, these were 93.90±0.58, respectively. The overall performance metrics showed negligible variability across seeds. However, Table 2 and Table 3, and Figure 3 report the results obtained with random seed 2025.

Figure 3 shows the confusion matrix on subject level (left) and trial level (right). These matrices demonstrate strong classification performance across all three classes; however, the trial-level matrix presents exact number of misclassified trials for each class. The analysis from Figure 3 indicates that most misclassifications occurred between the AD and CJD classes, whereas the CNTRL class showed highly consistent classification performance. This behavior may reflect overlapping EEG characteristics and inter-subject variability between neurodegenerative conditions, which can make their discrimination more challenging compared to healthy controls.

### Edge-AI Deployment

To assess the deployability of the proposed EEG decoding framework in real-world BCI scenarios, we evaluated the performance of EEGDecoder by deploying on an edge-AI device. To this end, Jetson AGX Orin [20] was used as the target platform, with an emphasis on minimizing resource utilization, energy consumption, and inference latency, aligning with the goals of sustainable clinical deployment. It is to be noted that, in this work, the analysis mainly focuses on inference efficiency rather than training performance on the edge-AI device, as BCI systems typically require real-time or near-real-time decision making after model deployment. The best-performing model for each subject was exported to ONNX format, ensuring hardware-agnostic and optimized execution. Edge-AI device was configured with JetPack 6.0 (Ubuntu 22.04). Generally, the models are converted into ONNX format for cross-platform compatibility. The *ONNX*-based models were then used to measure EEG decoding performance and other quantitative metrics for Green AI and energy efficiency in a deployment-oriented environment, enabling direct comparison with GPU-based performance. The comparative results of decoding and inference time of proposed model on local GPU system and edge-AI (i.e., Jetson AGX Orin by NVIDIA (64 GB)) for class-level are reported in Table 3. The deployed model maintains the same classification performance despite operating under significantly stricter power and resource constraints. The overall accuracy remains the same at 97.08%, demonstrating that edge-AI deployment does not degrade predictive performance. The class-wise results show that the CNTRL class achieves perfect classification (100%), while the AD and CJD classes also exhibit robust performance. For each held-out subject, the total inference time was recorded and normalized by the number of test trials to obtain the average inference time per trial. As expected, inference latency on the edge device is higher than on the local GPU, with an average inference time of approximately 56.66 milliseconds (ms) per trial as compared to local GPU of 2.61 ms.

**Figure 4:**
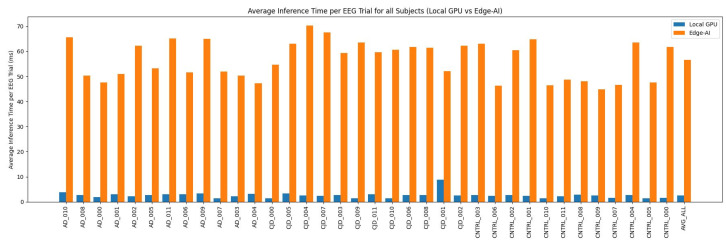
Average inference time per trial across left-out subjects. Comparison of total inference time per trial (normalized by the number of test trials) for each subject when processed on the edge device versus the local system.

Further, subject-wise average inference time per trial is shown in Figure 4; the x-axis shows the subjects and the y-axis the average inference time (ms) per trial. Blue bars denote the local GPU and yellow the edge-AI device.

Table 4 presents a comprehensive comparison of the proposed model across key computational and energy efficiency metrics on a local GPU and an edge-AI device. The model consists of 654.7 K parameters and requires approximately 210 million FLOPs (MFLOPs) per inference, indicating a lightweight architecture suitable for deployment in resource-constrained environments. On the local GPU, the model achieves a low inference latency of 2.61 ms, with a power consumption of 23.32 Watts (W), resulting in an energy cost of 0.0608 Joules (J) per inference. When deployed on the edge-AI device, the same model maintains identical computational complexity (parameters and FLOPs) while operating under significantly lower power consumption (4.16 W). Although the latency increases to 56.66 ms due to hardware constraints, the model remains within real-time processing limits for EEG applications. The corresponding energy consumption per inference is 0.2359 J, demonstrating efficient operation under low-power conditions. These results highlight a key trade-off between latency and power: the GPU achieves faster inference at higher power, whereas the edge device provides energy-efficient inference at lower power budgets. Importantly, the model retains consistent computational requirements across platforms, confirming its portability and deployment flexibility. Notably, the measured GPU power consumption (23 W) is close to the typical idle or low-utilization power range (10–20 W) reported for workstation-class GPUs such as the NVIDIA RTX 4000 Ada Generation, indicating that the proposed model imposes minimal additional computational load. In contrast, the edge-AI device operates at a substantially lower absolute power budget, reinforcing its suitability for energy-constrained environments.

## 4. Conclusions

This work presents a cross-subject EEG classification framework that explicitly addresses the challenges of inter-subject variability in Alzheimer’s disease classification. With a lightweight hybrid CNN–Transformer architecture evaluated on the LOSO mechanism and energy-efficient edge-AI deployment, the proposed approach achieves high classification accuracy while minimizing computational cost and power consumption. The results demonstrate the feasibility of deploying EEG-based diagnostic models in real-world clinical environments in a sustainable and environmentally responsible manner, paving the way for practical, scalable, and green AI-driven neurodiagnostic systems. Due to dataset anonymization protocols, detailed subject-level metadata such as age distribution, gender, medication status, disease severity, and other clinical variables were not accessible. Consequently, the analysis of cohort comparability and potential confounding factors could not be investigated comprehensively. Since this study provides preliminary evidence based on data collected from a single center, we acknowledge that broader generalization requires multi-center external validation. Additionally, expanding the experiment on a larger cohort of AD and CJD subjects will refine the utility of the proposed approach for real-world clinical environments. Further, feature importance analysis (e.g., attention heatmaps) can be performed to visualize the EEG features, as the transformer focuses on during classification, and it would strengthen the applicability of the model in clinical settings for better diagnosis.

## Figures and Tables

**Table 1:** Hyperparameters of the proposed hybrid CNN–Transformer model.

Hyperparameter	1D-CNN	Transformer Encoder
Learning Rate	0.001	
Batch Size	64	
Optimizer	Adam	
Weight Decay	$1\times 10^{-4}$	
Epochs	30	
Activation Function	ELU	ReLU (FFN)
Dropout Rate	–	Default (Transformer)
Number of 1D-CNN Layers	2	–
Kernel Sizes	Conv1 = 25, Conv2 = 15	–
Pooling Size	4, 4	–
Encoder Layers	–	2
Attention Heads	–	2
Embedding Dimension ($d_{\mathrm{model}}$)	–	64
Sequence Length ($T^{\prime }$)	–	75

**Table 2:** Model performance on LOSO evaluation mechanism averaged across 36 subjects (metrics in percentage, and Cohen’s Kappa in standard format).

Model	Accuracy	Precision	Recall	F1-Score	Cohen’s Kappa
EEGDecoder	97.08	94.22	93.45	93.83	0.95
EEGNet	93.90	88.72	85.45	86.70	0.87

**Table 3:** Comparison of proposed model on Local GPU and Edge-AI (Jetson AGX Orin) for accuracy (in percentage) and inference time (millisecond).

Class	Local System (NVIDIA GPU)		Edge-AI (Jetson AGX Orin)	
	Accuracy	Time (ms)	Accuracy	Time (ms)
AD	91.66	2.94	91.66	55.07
CJD	99.58	2.61	99.58	61.34
CNTRL	100.00	2.28	100.00	53.50
Overall	97.08	2.61	97.08	56.66

**Table 4:** Performance comparison of proposed model across different quantitative metrics of energy efficiency between local GPU and edge-AI device.

Device	Framework	Params	FLOPs	Latency (ms)	Power (W)	Energy (J)
GPU	PyTorch	654.7 K	210 M	2.61	23.32	0.0608
Jetson	ONNX	654.7 K	210 M	56.66	4.16	0.2359

## Data Availability

The analyzed dataset is available at the following link: https://github.com/AI-Lab-UniRC/FAIR-NAEL-Database (accessed on 7 April 2026).
